# A phase III clinical trial on the immunogenicity of a tetanus vaccine, adsorbed in individuals aged 18 years and above in China

**DOI:** 10.3389/fimmu.2026.1901212

**Published:** 2026-08-19

**Authors:** Qiuyue Mu, Zhe Li, Xiaolong Li, Tian Feng, Yajun Tan, Xue Wang, Feiyu Wang, Yuanxue Gao, Xiuwen Sui, Peng Wan, Guoxia Dong, Shunda Zhang, Bingzhuo Shi, Jinbo Gou, Yuhang Jiao, Haitao Huang, Liyong Yuan, Ruizhi Zhang

**Affiliations:** 1 Guizhou Province Centers for Disease Control and Prevention, Guiyang, China; 2 National Institutes for Food and Drug Control, State Key Laboratory of Drug Regulatory Science, Beijing, China; 3 CanSino Biologics Inc., Tianjin, China

**Keywords:** immunogenicity, immunogenicity persistence, phase III clinical trial, positive control design, tetanus vaccine, adsorbed

## Abstract

**Objective:**

To evaluate the immunogenicity of a tetanus vaccine, adsorbed (TTVA) in people aged 18 years and above.

**Methods:**

A randomized, double-blinded, and positive controlled clinical trial was conducted in Bijie, Guizhou Province, China. Participants aged 18 years and above were recruited and randomly assigned 1:1 to receive the experimental (TTVA) or control tetanus vaccine, adsorbed (TT) vaccine. Serum samples were collected from all participants before vaccination, 30 days after vaccination, and 6 months after vaccination in the first 240 participants to detect tetanus toxoid IgG antibody levels.

**Results:**

At 30 days after vaccination, the geometric mean concentration (GMC) of antibodies in the experimental group and the control group were 3.205 and 2.387 (*P* = 0.0012), the seroconversion rates were 89.07% and 87.47% (*P* = 0.6149), the seropositivity were 98.38% and 98.79% (*P* = 0.4850), and the geometric mean increase (GMI) were 35.529 and 27.604 (*P* = 0.0026), respectively. Six months after vaccination, the GMCs of antibodies in the experimental group and the control group were 1.025 and 0.803, respectively (*P* = 0.2119), the seropositivity were 98.25% and 95.73%, respectively (*P* = 0.4008), and the GMIs were 9.466 and 7.661, respectively (*P* = 0.2106).

**Conclusion:**

TTVA vaccine has good immunogenicity in people aged 18 years and above.

**Clinical trial registration:**

https://clinicaltrials.gov, identifier NCT06120751.

## 1 Introduction

Tetanus is an acute, often fatal infectious disease caused by the neurotoxin of *Clostridium tetani*, a spore-forming anaerobic bacterium ubiquitously present in soil, dust, and animal feces. The pathogen typically enters the human body through contaminated wounds, puncture injuries, or umbilical stump infections in newborns. Once inside a suitable anaerobic environment, the bacteria proliferate and produce tetanospasmin, a potent neurotoxin that blocks inhibitory neurotransmitter release in the central nervous system, leading to the characteristic clinical manifestations of trismus, generalized muscle rigidity, autonomic instability, and paroxysmal spasms (1, 2). The disease affects individuals across all age groups. Without medical intervention, the case fatality rate approaches 100%, particularly among neonates and the elderly (3, 4). Even with modern intensive care, the mortality rate remains between 30% and 50% globally, reflecting the severe pathophysiological impact and the persistent challenges in management (5, 6). Despite being entirely preventable through vaccination, tetanus remains a significant public health issue in many low- and middle-income countries where immunization coverage is inadequate. Meanwhile, there are still rare cases of the disease among unvaccinated or inadequately vaccinated adults in high-income countries (7).

China has made remarkable progress in tetanus control since the introduction of diphtheria-tetanus-pertussis (DTP) vaccine into the national immunization program in 1978, achieving nationwide coverage by 1998. As a result, neonatal tetanus—designated as a Class B notifiable infectious disease—has been eliminated since 2012 (8). However, the epidemiology of adult tetanus in China presents a contrasting picture. Due to the absence of a mandatory reporting system for non-neonatal tetanus, the true incidence is likely underestimated. A survey in Guilin City from 2015 to 2017 showed that the incidence of post-traumatic tetanus was 0.43/100,000, and the survey results in Shijiazhuang and Nanjing City from 2014 to 2018 showed that the incidence of post-traumatic tetanus was 0.42/100,000 and 0.17/100,000, respectively, and the average annual incidence in the population over 65 years of age was 1.32/100,000 and 0.17/100,000, respectively, which was much higher than the incidence of post-traumatic tetanus in the United States and Europe (9). Besides, a recent systematic literature review covering 2000 to 2022 identified 6,084 adult tetanus cases across China, with 83.42% occurring in individuals over 40 years of age, highlighting the disproportionate burden in middle-aged and older adults (10). Population-based serosurveys corroborate these clinical findings: a study in Fujian Province demonstrated that tetanus antibody seropositivity rates declined significantly with age, with only 39.2% of individuals aged 40–60 years maintaining protective antibody levels (≥0.1 IU/mL) (11). These data suggested that a substantial proportion of Chinese adults are inadequately protected against tetanus and remain vulnerable to infection following injury.

Currently, the Chinese immunization schedule mandates three primary doses of tetanus-containing vaccines (TTCVs) by six months of age, followed by booster doses at 18–24 months and again at six years. No routine tetanus booster vaccinations are recommended after the age of six years, leaving adolescents and adults without scheduled re-immunization. This gap is particularly concerning given that antibody levels induced by a complete primary series are generally maintained for 5 to 10 years and tend to wane over time, especially in older adults (12). In contrast, many high-income countries have adopted routine booster strategies, administering TTCVs every 10 to 20 years throughout adulthood, in alignment with WHO recommendations (13). In 2024, China issued the “Expert consensus on the use of tetanus vaccines for the high-risk group of post-traumatic tetanus”, signaling a paradigm shift toward proactive adult immunization to sustain protective immunity (9). In this study, the tetanus vaccine, adsorbed (TTVA) evaluated in this study was developed by Cansino Biological Co., Ltd. The study was designed to assess the immunogenicity of TTVA in individuals aged 18 years and above, with a focus on short-term antibody responses and immune persistence at six months post-vaccination. The results are expected to inform the potential inclusion of TTVA in China’s adult tetanus immunization strategies.

## 2 Materials and methods

### 2.1 Study design

This is a randomized, double-blinded and positive controlled trial studied in Bijie, Guizhou Province. Eligible participants aged 18 years and above were enrolled from 20 March 2024 to 29 March 2024. A total of 1,000 participants were enrolled and randomly assigned in a 1:1 ratio to receive a single dose of TTVA or the control tetanus vaccine, adsorbed (TT) vaccine, with a block size of 10. The first 240 enrolled participants (120 per group) were included in the 6-month immunogenicity persistence follow-up. Exclusion criteria included: (1) having been diagnosed with tetanus infection; (2) having received tetanus vaccine or vaccines containing tetanus toxoid antigen components within 5 years; (3) axillary temperature > 37.0 °C on the day of enrolment; (4) positive urine pregnancy test results or pregnancy and lactation in women; (5) having a pregnancy plan or fertility plan during the study in women of childbearing age, male volunteers with partners of women of childbearing age; (6) having thrombocytopenia, any coagulation dysfunction or receiving anticoagulant therapy, which may cause contraindications to intramuscular injection; (7) having severe respiratory diseases, severe cardiovascular diseases, liver and kidney diseases, malignant tumors, severe infectious or allergic skin diseases, hypertension that cannot be controlled by drugs (systolic blood pressure ≥ 160 mmHg, diastolic blood pressure ≥ 100 mmHg), and being in acute or active chronic diseases within 7 days; (8) having a history of severe neurological diseases such as epilepsy, convulsions, and encephalopathy or psychiatric history; (9) History of previous vaccination or severe drug allergy (anaphylactic shock, allergic laryngeal edema, Henoch-Schonlein purpura, thrombocytopenic purpura, local allergic necrosis reaction (Arthus reaction), etc.); (10) allergy to a component of the vaccine used in this clinical trial (mainly including tetanus toxoid, aluminum hydroxide, sodium chloride, etc.) or previous vaccination allergy to the above components; (11) known or suspected immunological dysfunction (such as HIV infection, thyroid, pancreas, liver, spleen, kidney disease history or resection history), received immunosuppressive therapy, cytotoxic therapy, systemic glucocorticoid therapy (≥ 14 days) within 6 months before enrolment (excluding corticosteroid spray therapy for allergic rhinitis, surface corticosteroid therapy for acute non-complicated dermatitis and other local medication); received blood products (whole blood, component blood, immunoglobulin, etc.); (12) received attenuated live vaccine within 14 days, and received subunit vaccine or inactivated vaccine within 7 days; (13) Any condition that, in the judgment of the investigator, may affect the assessment.

Since the study vaccines’ inner package were different, personnel responsible for vaccine preparation and administration are unblinded. Before enrolment, they signed confidentiality agreements to ensure that unblinded reports and documents are shared only with unblinded personnel and are properly stored in a secure area accessible only to authorized personnel. Unblinded personnel did not participate in any blinded on-site work. Except them, participants, other investigators, study statistician, and laboratory personnel are all blinded.

About 3–4 ml of blood samples were collected from all participants before vaccination, 30 days after vaccination, and 6 months after vaccination in the first 240 participants to detect tetanus toxoid IgG by Enzyme-linked immunosorbent assays (ELISA). The primary immunogenicity endpoint was the seroconversion rate of anti-tetanus toxoid IgG antibodies at 30 days after vaccination. The secondary immunogenicity endpoints included geometric mean increase (GMI), positivity rate, geometric mean concentration (GMC) of antibodies, and proportion of participants with antibody concentrations ≥ 1.0 IU/ml 30 days after vaccination. This study has been reviewed by the Ethics Committee of Center of Disease Control and Prevention of Guizhou Province and registered on the clinical trial platform (NCT06120751). Written informed consent was obtained prior to screening.

### 2.2 Study vaccines

Experimental vaccine was TTVA manufactured by Cansino Biological Co., Ltd., batch number TTVA 202303003, valid until March, 2025. Each 0.5 mL dose has a potency of ≥40 IU of tetanus toxoid, adsorbed onto aluminum hydroxide adjuvant. The vaccine is produced using an animal-origin-free culture medium and chromatography-based purification. Control vaccine was the TT manufactured by Chengdu Oulin Biotechnology Co., Ltd. (batch number 2T20230104, valid until January, 2026). Each 0.5 mL dose has a potency of ≥40 IU of tetanus toxoid, adsorbed onto aluminum hydroxide adjuvant. All study vaccines were stored and transported at 2-8 °C and used within expiry. The route and schedule of administration was a single intramuscular injection into the lateral deltoid muscle of the upper arm. All vaccines for study use have passed the inspection by the National Institutes for Food and Drug Control.

### 2.3 Laboratory testing

Anti-tetanus toxoid IgG antibodies were quantified using an indirect ELISA. Purified tetanus toxoid was used as the coating antigen. Blank microplates were manually coated with 100 μL per well of antigen at a concentration of 5 Lf/mL and incubated overnight at 4 °C. After five washes with 15-second intervals between each wash, pre-immunization serum samples were diluted 1:20 and post-immunization samples were diluted 1:1000. Both samples and the national anti-tetanus toxoid IgG standard reference were subjected to two-fold serial dilution. The plates were incubated at 37 °C for 1 hour and washed repeatedly. HRP-conjugated goat anti-human IgG secondary antibody (working concentration: 0.0002 mg/mL) was added at 100 μL per well and incubated at 37 °C for 1 hour. After washing, TMB substrate solution (100 μL per well) was added and incubated at room temperature in the dark for 10 minutes. The reaction was terminated with 50 μL of stop solution per well. Optical density was measured at 450 nm with a background correction wavelength of 630 nm. Antibody concentrations (mIU/mL) were calculated using a four-parameter logistic parallel line model by comparison with the serially diluted standard reference. The limit of quantification (LOQ) of the assay was 0.14 mIU/mL.

### 2.4 Statistical analysis

Sample size was calculated based on the IgG seroconversion rate 30 days after vaccination. According to the research (14), the IgG seroconversion rate 30 days after administration of the control vaccine was 99.8%. A conservative estimate suggests that the IgG seroconversion rate 30 days after vaccination will be no less than 95%. Assuming the difference in seroconversion rates between the experimental vaccine and the control vaccine is 0, with a non-inferiority margin of -5%, α = 0.025, and a power of 90%, and with participants assigned to the experimental and control groups in a 1:1 ratio, the Non-Inferiority Tests for the Difference Between Two Proportions method in PASS 23.0 calculates the sample size for each group to be approximately 400 subjects. With an estimated dropout rate of 10%, the sample size for each group is 445 participants. In accordance with the sample size requirements for safety in clinical trials of vaccines in China, the sample size for the experimental group should generally be no less than 500 participants. Therefore, the sample size has been expanded to 500 participants per group, for a total of 1,000 participants. The sample size for the persistence analysis was calculated based on the equivalence of antibody decline between the two vaccines over 6 months. Based on long-term follow-up studies of vaccines such as influenza and pneumococcal vaccines, the standard deviation of the annual slope of log-transformed antibody concentration decline is typically between 0.1 and 0.3. Taking a midpoint estimate of σ_slope_ = 0.23, an equivalence margin (log-scale) of Δ = 0.1, α = 0.05 (two-sided), power 1−β = 0.9, and a 1:1 allocation ratio, the calculated sample size was 112 participants per group. N = [2 × (Z_1-α/_2__ + Z_1-β_)^2^ × σ^2^_slope_]/Δ². Accounting for approximately 5% dropout, it was planned for 120 participants per group, totaling 240 participants.

SAS 9.4 software was used for statistical analyses. The 95% confidence intervals for the percentages will be calculated using Clopper-Pearson method. GMC and GMI will also use the following descriptive statistics: geometric means and 95% confidence intervals. GMCs were obtained by performing an inverse log transformation of the means of the log-transformed raw concentrations. The 95% confidence interval was obtained by using *t* distribution method to construct the 95% confidence interval of the mean of the logarithmic original concentration, and then the upper and lower limits of this confidence interval were reverse logarithmically transformed, so as to obtain the 95% confidence interval of the concentration at the original scale. The analytical methods for GMI are similar. Antibody positive criteria (protection criteria) are post-vaccination antibody concentration ≥ 0.1 IU/ml; antibody seroconversion criteria are pre-vaccination antibody concentration < 0.1 IU/ml, post-vaccination antibody concentration ≥ 0.4 IU/ml; or pre-vaccination antibody concentration ≥ 0.1 IU/ml, post-vaccination antibody 4-fold and above increase.

## 3 Results

### 3.1 Baseline demographic characteristics

**Figure 1:**
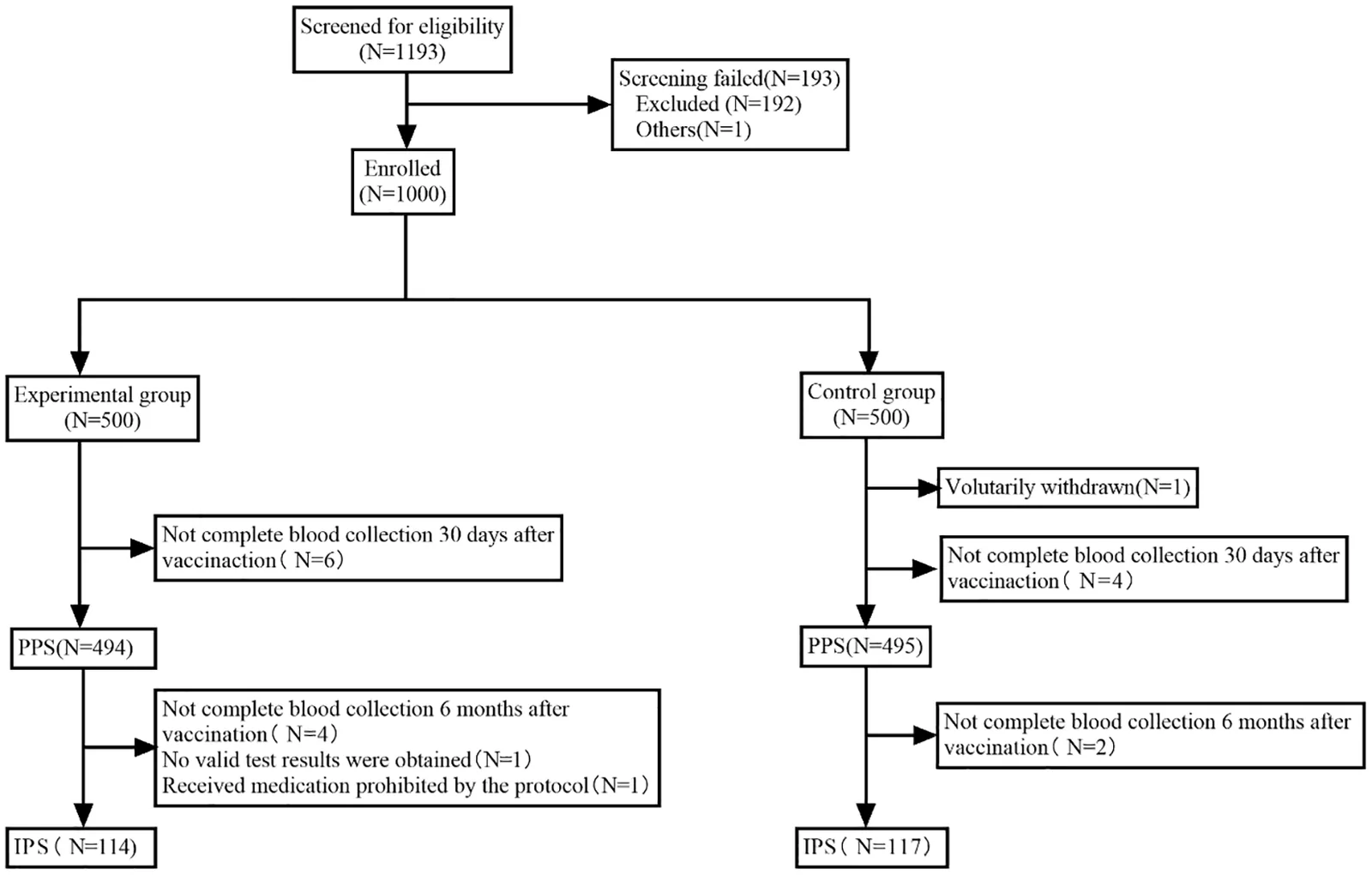
Screening and enrolment process. PPS, Per Protocol Set. IPS, Immunogenicity Persistence Set.

A total of 1,193 volunteers were screened in this study, 193 were screening failures, and 1000 participants were finally enrolled, with 500 in each of the experimental and control groups. There were 494 participants who completed blood collection in experimental group and 495 participants who completed blood collection in control group 30 days after vaccination. The persistence subset comprised the first 240 participants enrolled in chronological order. Experimental and control groups included 114 and 117 participants for immunogenicity persistence analysis (**Figure 1**). At 30 days and 6 months after vaccination, there was no statistical difference between the experimental group and the control group at all ages, while there was a slight difference in the gender at 6 months after vaccination in participants aged ≥ 46 years (**Table 1**).

**Table 1:** Demographic characteristics.

Characteristics	30 days after vaccination			6 months after vaccination		
	Experimental group	Control group	*P* value	Experimental group	Control group	*P* value
Age (years, n, $\bar{\text{X}}$)						
All Populations	494 (46.7)	495 (47.1)	0.6395	114 (45.5)	117 (47.6)	0.2240
18–45 years	229 (33.7)	224 (33.9)	0.7946	61 (35.4)	53 (36.1)	0.5006
≥ 46 years	265 (57.9)	271 (58.0)	0.8354	53 (57.2)	64 (57.1)	0.9377
Gender (n, %)						
All populations						
Male	263 (53.24)	258 (52.12)	0.7248	64 (56.14)	60 (51.28)	0.4591
Female	231 (46.76)	237 (47.88)		50 (43.86)	57 (48.72)	
18–45 years						
Male	108 (47.16)	113 (50.45)	0.4844	31 (50.82)	32 (60.38)	0.3060
Female	121 (52.84)	111 (49.55)		30 (49.18)	21 (39.62)	
≥ 46 years						
Male	155 (58.49)	145 (53.51)	0.2451	33 (62.26)	28 (43.75)	0.0460
Female	110 (41.51)	126 (46.49)		20 (37.74)	36 (56.25)	

### 3.2 Immunogenicity

#### 3.2.1 General populations

Based on the data presented in **Tables 2**, **3**, the immune responses in the general population were comparable between the experimental and control groups across different time points. At pre-vaccination baseline, no significant differences were observed between the two groups in terms of GMC, positivity rate, or the proportion with antibody levels ≥1.0 IU/mL, indicating comparable initial immune status. At 30 days after vaccination, the experimental group demonstrated significantly higher GMC (3.205 vs. 2.387; *P* = 0.0012) and GMI (35.529 vs. 27.604; *P* = 0.0026) compared to the control group. However, no significant differences were found in seroconversion rates (*P* = 0.6149), positivity rates (*P* = 0.4850), or the proportion achieving antibody levels ≥1.0 IU/mL (*P* = 0.7112). At 6 months after vaccination, all immune parameters, including GMC, GMI, positivity rate, and the proportion with antibody levels ≥1.0 IU/mL, showed no statistically significant differences between the two groups (all *P* > 0.05).

**Table 2:** GMC, seroconversion rate and GMI antibody levels before and after vaccination in general populations.

Immune status/Group	GMC		Seroconversion			GMI	
	GMC (95% CI)	*P* value	Number of seroconversions	seroconversion rate (95% CI)	*P* value	Fold increase (95% CI)	*P* value
Pre-immune							
Experimental group (n=494)	0.090 (0.084-0.097)	0.3661	–	–	–	–	–
Control group (n=495)	0.086 (0.081-0.092)		–	–		–	
30 days after vaccination							
Experimental group (n=494)	3.205 (2.796-3.673)	0.0012	440	89.07 (85.98-91.68)	0.6149	35.529 (31.441-40.149)	0.0026
Control group (n=495)	2.387 (2.127-2.679)		433	87.47 (84.23-90.26)		27.604 (24.734-30.808)	
6 months after vaccination							
Experimental group (n=114)	1.025 (0.757-1.389)	0.2119	–	–	–	9.466 (7.341-12.206)	0.2106
Control group (n=117)	0.803 (0.632-1.021)		–	–		7.661 (6.165-9.520)	

**Table 3:** Positive rate and antibody level ≥ 1.0 IU/ml before and after vaccination in general populations.

Immune status/Group	Positive rate			Antibody ≥ 1.0 IU/ml		
	Number positive	Positivity Rate (95% *CI*)	*P* value	Number of cases	Proportion (95% *CI*)	*P* value
Pre-immune						
Experimental group (n=494)	233	47.17 (42.69-51.67)	0.4874	1	0.20 (0.01-1.12)	0.9809
Control group (n=495)	220	44.44 (40.01-48.95)		1	0.20 (0.01-1.12)	
30 days after vaccination						
Experimental group (n=494)	486	98.38 (96.83-99.30)	0.4850	388	78.54 (74.66-82.08)	0.7112
Control group (n=495)	489	98.79 (97.38-99.55)		379	76.57 (72.58-80.23)	
6 months after vaccination						
Experimental group (n=114)	112	98.25 (93.81-99.79)	0.4008	55	48.25 (38.79-57.80)	0.9805
Control group (n=117)	112	95.73 (90.31-98.60)		54	46.15 (36.90-55.61)	

#### 3.2.2 Age stratification analysis

**Tables 4**, **5** elicited the immune responses following vaccination exhibited distinct age-dependent patterns. In the 18–45 years subgroup, the experimental group demonstrated significantly higher immune responses at 30 days post-vaccination compared to the control group, with notable differences in GMC (7.057 vs. 3.805; *P* < 0.0001), GMI (66.784 vs. 39.812; *P* < 0.0001), and seroconversion rate (98.25% vs. 94.64%; *P* = 0.0374). However, no significant differences were observed between the two groups at 6 months post-vaccination across all parameters, including GMC (*P* = 0.1199), GMI (*P* = 0.2011), positive rate (*P* = 0.4649) and the proportion achieving antibody levels ≥1.0 IU/mL (*P* = 0.9119).

**Table 4:** GMC, seroconversion rate and GMI antibody levels before and after vaccination by age.

Age group/Immune status/Group	GMC		Seroconversion			GMI	
	GMC (95% CI)	*P* value	Number of seroconversions	Seroconversion Rate (95% CI)	*P* value	Fold increase (95% CI)	*P* value
18–45 years							
Pre-immune							
Experimental group (n=229)	0.106 (0.096-0.117)	0.1533	–	–	–	–	–
Control group (n=224)	0.096 (0.087-0.105)		–	–		–	
30 days after vaccination							
Experimental group (n=229)	7.057 (5.978-8.331)	< 0.0001	225	98.25 (95.59-99.52)	0.0374	66.784 (57.325-77.804)	< 0.0001
Control group (n=224)	3.805 (3.271-4.425)		212	94.64 (90.83-97.20)		39.812 (34.278-46.240)	
6 months after vaccination							
Experimental group (n=61)	2.140 (1.407-3.254)	0.1199	–	–	–	16.518 (11.497-23.731)	0.2011
Control group (n=53)	1.404 (1.023-1.928)		–	–		12.283 (9.412-16.029)	
≥ 46 years							
Pre-immune							
Experimental group (n=265)	0.079 (0.072-0.086)	0.8453	–	–	–	–	–
Control group (n=271)	0.080 (0.073-0.086)		–	–		–	
30 days after vaccination							
Experimental group (n=265)	1.620 (1.363-1.926)	0.9854	215	81.13 (75.89-85.66)	0.9012	20.594 (17.558-24.155)	0.9301
Control group (n=271)	1.623 (1.389-1.898)		221	81.55 (76.41-85.98)		20.395 (17.567-23.678)	
6 months after vaccination							
Experimental group (n=53)	0.440 (0.318-0.607)	0.5373	–	–	–	4.987 (3.792-6.560)	0.8546
Control group (n=64)	0.506 (0.369-0.693)		–	–		5.182 (3.824-7.022)	

**Table 5:** Seropositivity and antibody levels ≥ 1.0 IU/ml before and after vaccination by age.

Age group/Immune status/Group	Positive rate			Antibody ≥ 1.0 IU/ml		
	Number positive	Positivity Rate (95% CI)	*P* value	Number of cases	Proportion (95% CI)	*P* value
18–45 years						
Pre-immune						
Experimental group (n=229)	129	56.33 (49.64-62.85)	0.2093	1	0.44 (0.01-2.41)	1.0000
Control group (n=224)	113	50.45 (43.71-57.17)		1	0.45 (0.01-2.46)	
30 days after vaccination						
Experimental group (n=229)	229	100 (98.40-100.00)	0.4945	215	93.89 (89.96-96.62)	0.0774
Control group (n=224)	223	99.55 (97.54-99.99)		200	89.29 (84.48-93.01)	
6 months after vaccination						
Experimental group (n=61)	61	100 (94.13-100.00)	0.4649	42	68.85 (55.71-80.10)	0.9119
Control group (n=53)	52	98.11 (89.93-99.95)		37	69.81 (55.66-81.66)	
≥ 46 years						
Pre-immune						
Experimental group (n=265)	104	39.25 (33.33-45.41)	0.9950	0	0.00 (0.00-1.38)	1.0000
Control group (n=271)	107	39.48 (33.62-45.58)		0	0.00 (0.00-1.35)	
30 days after vaccination						
Experimental group (n=265)	257	96.98 (94.14-98.69)	0.3771	173	65.28 (59.22-71.00)	0.8514
Control group (n=271)	266	98.15 (95.75-99.40)		179	66.05 (60.08-71.67)	
6 months after vaccination						
Experimental group (n=53)	51	96.23 (87.02-99.54)	0.6879	13	24.53 (13.76-38.28)	0.8019
Control group (n=64)	60	93.75 (84.76-98.27)		17	26.56 (16.30-39.09)	

In the ≥46 years subgroup, no statistically significant differences were observed between the experimental and control groups at any time point. At 30 days post-vaccination, GMC (1.620 vs. 1.623; *P* = 0.9854), GMI (20.594 vs. 20.395; *P* = 0.9301), and seroconversion rates (81.13% vs. 81.55%; *P* = 0.9012) were comparable between groups. Similarly, at 6 months post-vaccination, all immune parameters, including GMC, GMI, positivity rates, and the proportion with antibody levels ≥1.0 IU/mL, showed no significant between-group differences (all *P* > 0.05). Notably, antibody responses in this older age group were substantially lower than those observed in the younger subgroup, with GMC values declining to below 0.506 in both groups by 6 months.

#### 3.2.3 Analysis by gender

According to the data shown in **Tables 6**, **7**, a gender-based analysis presented different patterns in immune response before and after vaccination. In the male subgroup, no significant differences were observed between the experimental and control groups at either time point. At 30 days after vaccination, GMC (2.649 vs. 2.391; *P* = 0.4312), seroconversion rate (85.17% vs. 88.37%; *P* = 0.2812), GMI (30.282 vs. 29.744; *P* = 0.8779), positivity rate (98.10% vs. 98.45%; *P* = 1.0000), and the proportion achieving antibody levels ≥1.0 IU/mL (73.76% vs. 77.52%; *P* = 0.3182) were all comparable between experimental and control groups. Similarly, at 6 months after vaccination, no significant differences were observed across all immune parameters (all *P* > 0.05).

**Table 6:** GMC, seroconversion rate and GMI antibody levels by gender.

Gender/Immune status/Group	GMC		Seroconversion			GMI	
	GMC (95% *CI*)	*P* value	Number of seroconversions	Seroconversion rate (95% *CI*)	*P* value	Fold increase (95% *CI*)	*P* value
Male							
Pre-immune							
Experimental group (n=263)	0.087 (0.079-0.097)	0.2225	–		–	–	–
Control group (n=258)	0.080 (0.073-0.088)		–	–		–	
30 days after vaccination							
Experimental group (n=263)	2.649 (2.176-3.225)	0.4312	224	85.17 (80.29-89.24)	0.2812	30.282 (25.523-35.930)	0.8779
Control group (n=258)	2.391 (2.032-2.814)		228	88.37 (83.82-92.02)		29.744 (25.516-34.672)	
6 months after vaccination							
Experimental group (n=64)	1.029 (0.677-1.564)	0.6147	–	–	–	9.259 (6.622-12.947)	0.8719
Control group (n=60)	0.896 (0.631-1.272)		–	–		8.923 (6.551-12.154)	
Female							
Pre-immune							
Experimental group (n=231)	0.093 (0.086-0.102)	0.9718	–	–	–	–	–
Control group (n=237)	0.094 (0.086-0.102)		–	–		–	
30 days after vaccination							
Experimental group (n=231)	3.980 (3.309-4.788)	< 0.0001	216	93.51 (89.52-96.32)	0.0117	42.619 (35.855-50.658)	< 0.0001
Control group (n=237)	2.382 (2.021-2.808)		205	86.50 (81.48-90.58)		25.450 (21.735-29.799)	
6 months after vaccination							
Experimental group (n=50)	1.020 (0.646-1.609)	0.2049	–	–	–	9.738 (6.506-14.573)	0.1121
Control group (n=57)	0.716 (0.512-1.000)		–	–		6.525 (4.790-8.888)	

**Table 7:** Positive rate and antibody level ≥ 1.0 IU/ml after vaccination analyzed by gender.

Gender/Immune status/Group	Positive rate			Antibody ≥ 1.0 IU/ml		
	Numbers of positive rate	Positive rate (95% *CI*)	*P* value	Number of cases	Proportion (95% *CI*)	*P* value
Male						
Pre-immune						
Experimental group (n=263)	116	44.11 (38.01-50.34)	0.1838	1	0.38 (0.01, 2.10)	1.0000
Control group (n=258)	99	38.37 (32.41, 44.61)		1	0.39 (0.01, 2.14)	
30 days after vaccination						
Experimental group (n=263)	258	98.10 (95.62-99.38)	1.0000	194	73.76 (68.01-78.98)	0.3182
Control group (n=258)	254	98.45 (96.08-99.58)		200	77.52 (71.93-82.46)	
6 months after vaccination						
Experimental group (n=64)	63	98.44 (91.60-99.96)	0.3532	32	50.00 (37.23-62.77)	0.8528
Control group (n=60)	57	95.00 (86.08-98.96)		31	51.67 (38.39-64.77)	
Female						
Pre-immune						
Experimental group (n=231)	117	50.65 (44.01-57.27)	0.9301	0	0.00 (0.00-1.58)	1.0000
Control group (n=237)	121	51.05 (44.50-57.58)		0	0.00 (0.00-1.54)	
30 days after vaccination						
Experimental group (n=231)	228	98.70 (96.25-99.73)	0.6822	194	83.98 (78.60-88.46)	0.0230
Control group (n=237)	235	99.16 (96.99-99.90)		179	75.53 (69.54-80.86)	
6 months after vaccination						
Experimental group (n=50)	49	98.00 (89.35-99.95)	1.0000	23	46.00 (31.81-60.68)	0.5559
Control group (n=57)	55	96.49 (87.89-99.57)		23	40.35 (27.56-54.18)	

In contrast, the female subgroup exhibited significantly higher immune responses in the experimental group compared to the control group at 30 days after vaccination. The experimental group showed markedly elevated GMC (3.980 vs. 2.382; *P* < 0.0001), greater GMI (42.619 vs. 25.450; *P* < 0.0001), and a higher seroconversion rate (93.51% vs. 86.50%; *P* = 0.0117). Additionally, the proportion of females achieving antibody levels ≥1.0 IU/mL was significantly higher in the experimental group (83.98% vs. 75.53%; *P* = 0.0230). However, at 6 months after vaccination, no significant differences remained between the two groups in females for any immune parameter, including GMC (*P* = 0.2049), GMI (*P* = 0.1121), positivity rate (*P* = 1.0000), and the proportion with antibody levels ≥1.0 IU/mL (*P* = 0.5559).

## 4 Discussion

In this phase III randomized, double-blinded, positive-controlled trial, the novel TTVA demonstrated robust immunogenicity in adults aged 18 years and above. At 30 days post-vaccination, the TTVA group achieved significantly higher GMC and GMI compared to the control group, although seroconversion and seropositivity rates were comparable. At six months post-vaccination, immune responses remained substantial with no significant between-group differences, indicating durable persistence. These findings suggest that TTVA elicits a strong and sustained immune response, particularly in younger adults and females. The superior GMC and GMI observed in the TTVA group at day 30 likely reflect the vaccine’s advanced manufacturing characteristics. Unlike conventional tetanus toxoid vaccines, TTVA is produced using an animal-origin-free culture medium, which eliminates the risk of adventitious virus contamination. The toxoid is purified via chromatography, achieving higher purity than comparable products. Furthermore, the aluminum hydroxide adjuvant undergoes a post-crystallization treatment that yields uniformly sized particles (<10 µm) with a larger specific surface area, enhancing antigen adsorption and potentially improving immunogenicity. These features may explain the higher antibody levels achieved by TTVA, particularly in the early post-vaccination period.

The age-stratified results revealed markedly different immune profiles between younger and older adults. In the 18–45 years subgroup, the TTVA group showed significantly higher GMC and GMI at day 30, with a seroconversion rate of 98.25% versus 94.64% (*P* = 0.0374). These values compare favorably with a recently licensed tetanus vaccine, which reported a day-30 GMC of 4.721 IU/mL and a long-term protection rate (≥1.0 IU/mL) of 92.77% in adults aged 18–44 years (15). Notably, our study extended to participants up to 81 years of age, providing immunogenicity data for older adults who are often underrepresented in vaccine trials. In contrast, the ≥46 years subgroup exhibited substantially attenuated responses. Day-30 GMC values were approximately 1.62 IU/mL in both groups, with no significant differences, and by six months, GMC declined to below 0.506 IU/mL. This age-related decline is consistent with immunosenescence, which impairs both the magnitude and durability of vaccine-induced immunity (16). Older adults also frequently present with comorbidities such as hypertension, diabetes, and cardiovascular disease, which further compromise immune function and increase the risk of severe tetanus outcomes (16). These findings underscore the need for tailored immunization strategies in older populations, including potentially higher antigen doses or more frequent booster schedules. The gender-based analysis showed that the superior immunogenicity of TTVA at day 30 was driven primarily by female participants. In females, TTVA elicited significantly higher GMC, GMI, seroconversion rate, and proportion with antibody levels ≥1.0 IU/mL. This result may be attributed to age distribution. In the TTVA group, 52.4% of female participants were aged 18–45 years, compared to 46.8% in the control group, with a higher proportion of older females (≥46 years) in the control group. Since younger adults (18–45 years) mounted significantly higher antibody responses than older adults (≥46 years), the age distribution difference likely confounded the gender comparison (17). Additionally, the small sample size in the 6-month persistence subgroup may have further contributed to this confounding effect and limited the statistical power for robust subgroup comparisons.

The persistence data at six months is reassuring. In the 18–45 years subgroup, the TTVA group maintained a GMC of 2.140 IU/mL, and nearly 70% of participants retained antibody levels ≥1.0 IU/mL—well above the protective threshold of 0.1 IU/mL. This suggested that a single dose of TTVA can provide durable protection for at least six months in younger adults, supporting its use as a booster in populations with waning immunity. In older adults, however, the rapid decline in antibody levels highlights the need for closer monitoring and potentially earlier revaccination.

There are some limitations in this study. First, this manuscript focuses exclusively on immunogenicity outcomes. Safety data including adverse reactions, adverse events and serious adverse events in experimental and control groups were not shown. While these data were fully collected and are currently under analysis, and will be reported later. Second, the study was conducted at a single site in Guizhou Province, limiting geographical generalizability. Multi-center and large-sample trials may be conducted across diverse populations according to further needs. Third, the follow-up duration was only six months, insufficient to characterize long-term antibody persistence or immune memory recall. Extended follow-up of at least 2–5 years is necessary to determine the optimal booster interval. Finally, the subgroup analyses by age and gender were exploratory and were not adjusted for multiple comparisons, which may increase the risk of Type I error. A baseline gender imbalance was observed within the ≥46-year subgroup at the 6-month time point, and no covariate adjustment was performed. Therefore, findings from these subgroup analyses should be interpreted with caution. Despite these limitations, this study included a wide age range (18–81 years) enables clinically relevant age-stratified analyses. The use of a licensed active control vaccine allows direct comparison with an existing standard of care.

In conclusion, TTVA demonstrates good immunogenicity in adults aged 18 years and above, inducing rapid, potent, and persistent antibody responses. The vaccine elicited superior GMC and GMI compared to a licensed control vaccine at day 30, particularly in younger adults and females, with comparable immune persistence at six months. Given the substantial burden of adult tetanus in China and the absence of routine adult booster vaccination, TTVA represents a promising new option for tetanus prevention. These findings support further evaluation of TTVA in multi-center trials with extended follow-up and integrated safety monitoring, and ultimately its potential inclusion in China’s adult immunization strategies.

## Data Availability

The raw data supporting the conclusions of this article will be made available by the authors, without undue reservation.
